# Hyperthermia Stimulates HIV-1 Replication

**DOI:** 10.1371/journal.ppat.1002792

**Published:** 2012-07-12

**Authors:** Ferdinand Roesch, Oussama Meziane, Anna Kula, Sébastien Nisole, Françoise Porrot, Ian Anderson, Fabrizio Mammano, Ariberto Fassati, Alessandro Marcello, Monsef Benkirane, Olivier Schwartz

**Affiliations:** 1 Institut Pasteur, Unité Virus et Immunité, Département de Virologie, Paris, France; 2 CNRS, URA3015, Paris, France; 3 Université Paris Diderot, Sorbonne Paris Cité, Cellule Pasteur, Paris, France; 4 Institut de Génétique Humaine, Laboratoire de Virologie Moléculaire, Montpellier, France; 5 CNRS, UPR1142, Montpellier, France; 6 Laboratory of Molecular Virology, International Centre for Genetic Engineering and Biotechnology (ICGEB), Trieste, Italy; 7 Institut Pasteur, Unité de Virologie Moléculaire et Vaccinologie, Paris, France; 8 Wohl Virion Centre, Division of Infection and Immunity, MRC Centre for Medical & Molecular Virology, University College London, London, United Kingdom; 9 INSERM U941, Hôpital Saint Louis, Paris, France; 10 Université Paris Diderot, Sorbonne Paris Cité, IUH, UMRS 941, Paris, France; NIH/NIAID, United States of America

## Abstract

HIV-infected individuals may experience fever episodes. Fever is an elevation of the body temperature accompanied by inflammation. It is usually beneficial for the host through enhancement of immunological defenses. In cultures, transient non-physiological heat shock (42–45°C) and Heat Shock Proteins (HSPs) modulate HIV-1 replication, through poorly defined mechanisms. The effect of physiological hyperthermia (38–40°C) on HIV-1 infection has not been extensively investigated. Here, we show that culturing primary CD4+ T lymphocytes and cell lines at a fever-like temperature (39.5°C) increased the efficiency of HIV-1 replication by 2 to 7 fold. Hyperthermia did not facilitate viral entry nor reverse transcription, but increased Tat transactivation of the LTR viral promoter. Hyperthermia also boosted HIV-1 reactivation in a model of latently-infected cells. By imaging HIV-1 transcription, we further show that Hsp90 co-localized with actively transcribing provirus, and this phenomenon was enhanced at 39.5°C. The Hsp90 inhibitor 17-AAG abrogated the increase of HIV-1 replication in hyperthermic cells. Altogether, our results indicate that fever may directly stimulate HIV-1 replication, in a process involving Hsp90 and facilitation of Tat-mediated LTR activity.

## Author Summary

Fever is a complex reaction triggered in response to pathogen infection. It induces diverse effects on the human body and especially on the immune system. The functions of immune cells are positively affected by fever, helping them to fight infection. Fever consists in a physiological elevation of temperature and in inflammation. While the role of inflammatory molecules on HIV-1 replication has been widely studied, little is known about the direct effect of temperature on viral replication. Here, we report that hyperthermia (39.5°C) boosts HIV-1 replication in CD4+ T cells. In single-cycle infection experiments, hyperthermia increased HIV-1 infection up to 7-fold. This effect was mediated in part by an increased activation of the HIV-1 promoter by the viral protein Tat. Our results also indicate that hyperthermia may help HIV-1 to reactivate from latency. We also show that the Heat Shock Protein Hsp90, which levels are increased at 39.5°C, mediates in a large part the positive effect of hyperthermia on HIV-1 infection. Our work suggests that in HIV-1-infected patients, fever episodes may facilitate viral replication.

## Introduction

Fever is a physiological process induced by endogenous pyretics (IL-6, IL-1β, TNFα) in response to stresses such as pathogen infection. It consists in hyperthermia, an elevation of the body temperature to 38–40°C, associated with an inflammatory state. Fever is generally beneficial for the host, triggering multiple events that lead to the strengthening of immunological defenses. For instance, hyperthermia increases dendritic cells (DC) maturation, migration and antigen presentation [1]. Hyperthermia positively impacts cytokine and antibody production by lymphocytes, and enhances their migration to lymph nodes [2], [3]. Hyperthermia also intensifies cytotoxic activity of Natural Killer cells and phagocytosis by macrophages [4], [5]. Together, these events explain why fever is often associated with better disease outcome [2].

Temperature has various consequences on viral replication. Infection at 41°C inhibits the replication of some human viruses such as Poliovirus, Herpes Simplex Virus type 1 and Measles Virus [6]. Heat shock inhibits Vesicular Stomatitis Virus and Mayaro Virus replication [7], [8]. In contrast, hyperthermia promotes infection by Rotavirus, Dengue Virus, Epstein-Barr Virus, Human Cytomegalovirus and plant viruses [9], [10], [11], [12], [13].

HIV-1-infected patients can experience fever at various stages of the disease. During acute infection, HIV-1 replication is intense, viral loads reach very high levels, and patients are subjected to fever and strong inflammation. Opportunistic infections, which are frequent at the final stages of AIDS, can also induce fever. They directly impact HIV-1 replication, and treating them significantly reduces viral loads [14]. Several millions of HIV-1-positive patients, the majority of which not receiving any treatment, also suffer from tuberculosis or malaria [15], [16]. The two causative pathogens induce fever episodes, and are associated with increased HIV-1 viral loads [17], [18], [19], [20], [21]. Fever may thus modify the environment for HIV-1 replication, either in a positive or a negative way. The relative contribution of direct effects of co-infecting pathogens, inflammation, and elevated temperature to this process is not clearly understood. The role of inflammation on HIV-1 pathogenesis has been widely documented [22], [23], [24], [25]. Inflammation and immune activation represent a driving force for CD4+ T cell depletion, facilitation of viral replication, and AIDS progression [23], [24], [25]. Immune activation also likely impacts the establishment of viral persistence [26]. In culture, pro-inflammatory cytokines such as IL-1β, IL-6 and Tumor Necrosis Factor α (TNFα) favor HIV-1 replication [27], [28], [29], [30]. Knowledge about the role of temperature on HIV-1 replication remains fragmented. Previous research has mainly been focused on heat shock, a transient and non-physiological treatment (a few minutes to a few hours) at 40–45°C, rather than on hyperthermia, an incubation at 38–40°C for up to a few days. Heat shock stimulates HIV-1 production in latently infected cell lines [31], [32] and in Peripheral Blood Mononucleated Cells (PBMCs) [33]. An increased temperature also enhances plasma membrane fluidity and might facilitate viral entry [34]. A precedent work, aimed at characterizing thermosensitive HIV-1 integrase mutants, did not report any viral increase at 7 days post infection (p.i.), at 39.5°C, using a high Multiplicity of Infection (MOI), in CEM cells [35].

Heat Shock Proteins (HSPs) are induced in response to stresses and act as chaperones, helping the folding of proteins and preventing their aggregation. HSPs are overexpressed in many types of tumoral cells, like multiple myeloma, breast or prostate cancer cells [36], [37], [38]. HSPs also have important roles in both innate and adaptive immunity [39]. Extracellular HSPs stimulate cytokine production by DCs. HSP-bound peptides can be endocytosed by DCs and other Antigen Presenting Cells through interaction with the CD91 receptor, and participate to antigen cross-presentation [39]. These properties led to the use of HSPs as adjuvants in vaccine development [40]. Several studies indicated that HIV-1 induces the synthesis of some HSPs such as Hsp27 or Hsp70 [41], [42]. Hsp70 is found in virions and might interfere with Vpr functions [42], [43], [44], [45], [46]. Hsp40 and Hsp70 have been suggested to regulate Nef activity [47]. Recently, it has been reported that Hsp90 and the transcription factor HSF-1 can both increase HIV-1 transcription [48], [49]. Hsp90 was also shown to rescue the impaired replication of ritonavir-resistant viruses [50]. In these studies, the role of HSPs in HIV-1 replication was mainly investigated through over-expression or depletion experiments, whereas the direct impact of temperature on HSPs levels and HIV-1 infection was not characterized. Overall, the importance of HSPs for HIV-1 replication is still not fully elucidated, and the precise role of temperature remains unknown.

We examined here the effect of physiological, fever-like temperature (39.5°C) on several steps of HIV-1 life cycle. We show that hyperthermia enhanced HIV-1 replication in primary CD4+ lymphocytes and cell lines. Transactivation of the Long Terminal Repeat (LTR) promoter by Tat, and viral reactivation in a model of latency, were both more potent at 39.5°C than at 37°C. Hyperthermia increased Hsp90 co-localization with actively transcribing HIV-1 provirus, suggesting a direct effect of this cellular protein on viral gene expression. Our study suggests that fever episodes may promote HIV-1 replication in infected individuals.

## Results

### Enhanced HIV-1 replication in CD4+ T cells grown at 39.5°C

**Figure 1:**
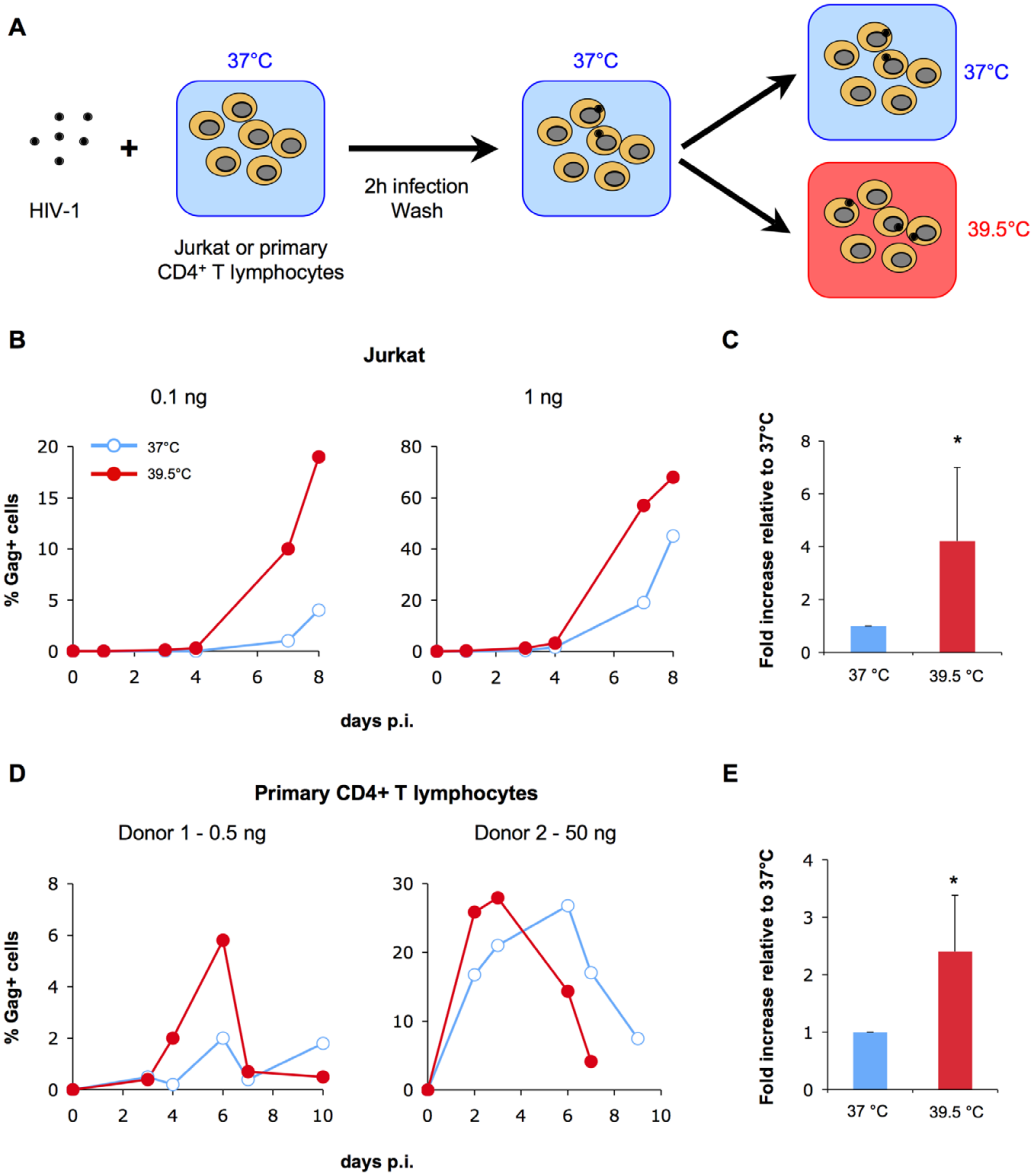
Hyperthermia enhances HIV replication. A: Experimental Outline. Jurkat or primary CD4+ T cells were infected with HIV-1 NL4-3 for 2 hours at 37°C and grown at either 37°C or 39.5°C for up to 10 days. B, D. Representative experiments with Jurkat and primary CD4+ T cells, respectively. The levels of intracellular Gag were measured by flow cytometry at the indicated time points. C, E: Mean ± SD of 5 independent experiments in Jurkat and primary CD4+ T cells, respectively. The area under the curve was calculated for all time points in Jurkat cells, and for time points occurring until the appearance of the peak at 39.5°C in primary CD4+ T cells. Statistical significance was assessed by the Wilcoxon test. p<0.05(*).

We first analyzed the effect of hyperthermia on HIV-1 replication in Jurkat lymphoid cells and in primary CD4+ T lymphocytes. After 2 hours of infection at 37°C, cells were cultivated either at 37°C or 39.5°C (Fig. 1A). Viral spread was then followed by measuring the appearance of Gag+ cells by flow-cytometry at different time points. A representative experiment in Jurkat cells, using different MOI (0.1 and 1 ng Gag p24/ml/10^6^ cells), indicates that HIV-1 replication was more rapid and efficient at 39.5°C than at 37°C (Fig. 1B). In a compilation of independent experiments, hyperthermia increased HIV-1 replication by 4 fold (Fig. 1C). In some experiments, when a higher MOI was used, the differences between the two temperatures were attenuated, probably because viral replication reached saturation levels at both 37°C and 39.5°C (not shown). Similar results were obtained upon infection of primary CD4+ T cells (Fig. 1D), even if the effect of hyperthermia was less marked (2.5 fold increase, Fig. 1E). Depending on the experiments, the peak of infection was either higher (Fig. 1D, donor 1) or occurred earlier (Fig. 1D, donor 2) under hyperthermic conditions. Thus, hyperthermia increases HIV-1 replication in both Jurkat and primary CD4+ T cells.

**Figure 2:**
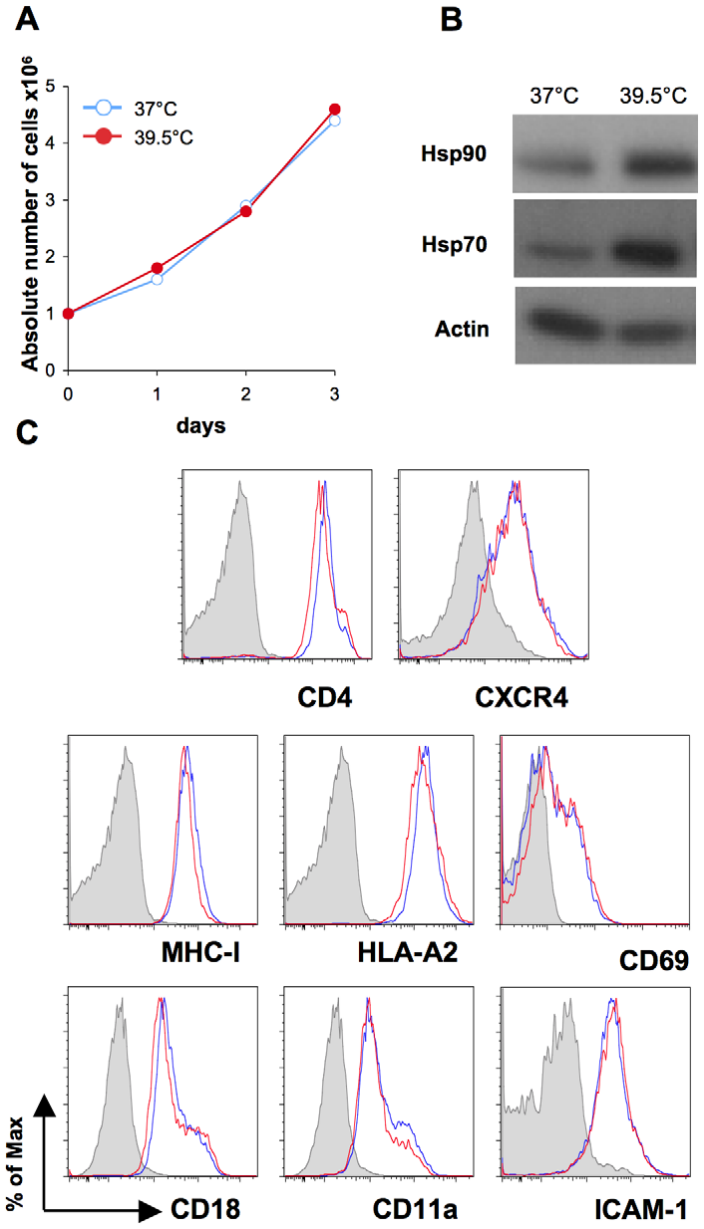
Cell growth and expression of Hsp70, Hsp90, and surface molecules in hyperthermic T cells. A: Primary CD4+ T cells were grown at 37°C or 39.5°C for 3 days. Cell growth was assessed every day by direct counting of living cells (Trypan blue exclusion). B: Primary CD4+ T cells were incubated 8 hours at 37°C or 39.5°C. Cell lysates were collected and probed by Western Blot for Hsp90, Hsp70 and Actin. C: Primary CD4+ T cells were grown at 37°C (blue line) or 39.5°C (red line) for 3 days. Cells were stained for the indicated surface markers and fixed in PFA. Isotype-matched monoclonal antibodies were used as negative controls (grey line).

We then examined how cells responded to hyperthermia in the absence of infection. The growth and viability of primary CD4+ T cells and Jurkat cells were not detectably influenced by hyperthermia (Fig. 2A and not shown). Hyperthermia significantly increased the amount of Hsp70 and Hsp90 in CD4+ T cells, as assessed by Western Blot (Fig. 2B). The surface levels of viral receptors (CD4 and CXCR4), Major Histocompatibility Complex class I (MHC-I) and HLA-A2, the activation marker CD69, and adhesion molecules ICAM-1 and LFA-1 (CD11a and CD18 chains), were similar at 37°C and 39.5°C (Fig. 2C). This suggests that hyperthermia does not profoundly alter the behavior of CD4+ T lymphocytes, with non-specific consequences on HIV-1 replication.

### Hyperthermia increases HIV-1 single-cycle infection but not lentivector transduction

**Figure 3:**
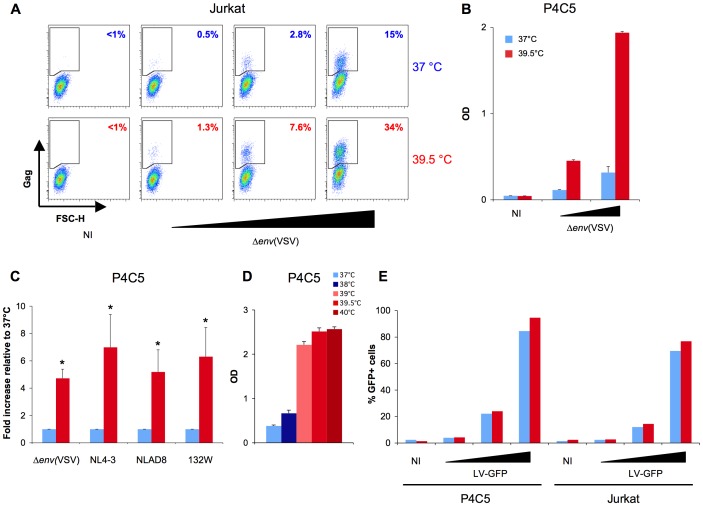
Hyperthermia increases infection with single-cycle HIV-1 but not with a GFP lentivector. A: Infection of Jurkat cells with single-cycle HIV. One representative experiment is shown. Cells were infected with 5, 20, or 100 ng Gag p24 of Δ*env*(VSV)/mL/10^6^ cells for 2 hours at 37°C or 39.5°C, washed, and grown at 37°C or 39.5°C. Gag levels were assessed by flow cytometry at 24 hours p.i. B: Infection of P4C5 cells with single-cycle HIV. One representative experiment is shown. 8×10^4^ P4C5 cells were plated in 96-well plates. Cells were infected in triplicates with 1 or 5 ng Gag p24 of Δ*env*(VSV) and grown at 37°C or 39.5°*C*. Infection was assessed 36 hours p.i. by measuring β-Galactosidase activity (570 nm OD). C: Mean ± SD of 4 independent experiments. P4C5 cells were infected with 1 ng Gag p24 of the indicated HIV strains. Statistical significance was assessed by the Mann-Withney test. p<0.05(*). D: Infection of P4C5 cells at different temperatures. One of two representative experiments is shown. Data are mean ± SD of triplicates. Using the same protocol as in B, cells were infected with 5 ng Gag p24 of NL4-3 and grown at the indicated temperatures. E: Transduction of P4C5 and Jurkat cells with a GFP expressing lentivector (LV-GFP). One of three representative experiments is shown. P4C5 or Jurkat cells were transduced with 1, 10 or 100 ng Gag p24/mL/10^6^ cells of LV-GFP. GFP levels were measured 24 hours later by flow cytometry.

We then asked if hyperthermia affected infection with single-cycle HIV-1. Jurkat cells were exposed to increasing doses of Δ*env*(VSV), a VSV-G-pseudotyped, *env-*deleted HIV-1 strain. Gag levels were assessed 24 hours later by flow-cytometry (Fig. 3A). At various viral inputs, the fraction of Gag+ cells was about 3 times greater at 39.5°C than at 37°C. Similar results were observed in P4C5 cells, a reporter HeLa cell line expressing CD4, CCR5, and harboring an HIV-1 LTR-*lacZ* cassette. Hyperthermia increased single-cycle infection with Δ*env*(VSV), assessed by measuring β-Galalactosidase activity (Fig. 3B) or Gag levels (not shown). We then tested a panel of viral strains with different tropisms. Hyperthermia increased infection by either the ×4 strain NL4-3, the R5 strain NLAD8, or the dual-tropic primary isolate 132W, by 4 to 7 fold in P4C5 cells (Fig. 3C). Therefore, the effect of hyperthermia is independent of the route of entry and of co-receptor usage. We then performed a temperature “titration” experiment. We infected P4C5 cells at temperatures ranging from 37°C to 40°C (Fig. 3D). Infection began to increase at 38°C (almost 2 fold), and was further enhanced at 39°C, 39.5°C and 40°C (6–7 fold).

In contrast, hyperthermia did not augment transduction of P4C5 and Jurkat cells with an HIV-1 lentivector (LV-GFP) (Fig. 3E). With this lentivector, GFP expression is driven by the CMV promoter, independently of Tat, suggesting that hyperthermia may induce its effects during or after HIV-1 transcription.

### Hyperthermia enhances Tat transactivation of the LTR

To gain further insight into the underlying mechanism of hyperthermia-mediated increased infection, we examined several steps of HIV-1 replication cycle. We first asked whether hyperthermia affects the half-life of cell-free viral particles after release. We incubated virions (produced at 37°C) at 37°C or 39.5°C for various periods of time (15 min to 24 hours). The infectivity of the viral preparations was then measured on P4C5 reporter cells. Incubation at 37°C led to a rapid drop of infectivity, with a half-life of about 2.5 hours. This half-life was slightly, but not significantly, decreased at 39.5°C (Fig. S1A).

**Figure 4:**
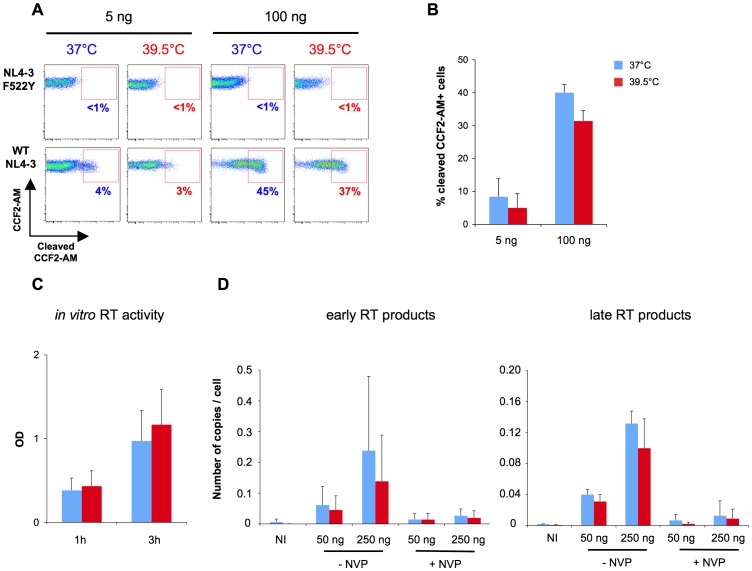
Hyperthermia does not stimulate viral fusion and reverse transcription. A: Viral fusion assay. One representative experiment is shown. Jurkat cells were exposed for 2 hours at 37°C or 39.5°C to 5 or 100 ng Gag p24/10^5^ cells/0.1 mL of either WT, or a non fusogenic *env* mutant (F552Y) NL4-3, both bearing the chimeric protein β-lactamase-Vpr. Viral access to the cytoplasm was assessed by flow cytometry, using the ability of β-lactamase to cleave CCF2-AM, a fluorogenic substrate. B: Mean ± SD of 3 independent experiments. C: In vitro assessment of RT activity. Mean ± SD of 2 independent experiments is shown. HIV-1 NL4-3 virions were lysed and incubated at 37°C or 39.5°C for 1 h or 3 h. RT activity was measured with the Innovagen RetroSys RT Activity Kit (405 nm OD). D: Viral DNA synthesis in Jurkat cells. Mean ± SD of 3 independent experiments is shown. Jurkat cells were infected with 50 ng or 250 ng Gag p24 of Δ*env*(VSV)//mL/10^6^ cells for 2 hours at 37°C or 39.5°C, washed, and grown at 37°C or 39.5°C. 8 hours p.i., cells were harvested and viral DNA was measured by quantitative PCR. As a control, the RT inhibitor nevirapine (NVP) was added during infection.

A previous study suggested that a higher temperature of infection enhances membrane fluidity and might thus facilitate viral entry [34]. To quantify HIV-1 entry in Jurkat cells, we used a virion fusion assay, which allows to discriminate cytoplasmic access of viral cores from endosomal capture [51], [52], [53]. This assay consists in the use of viruses containing a β-lactamase-Vpr (Blam-Vpr) protein chimera. The successful cytoplasmic access of Blam-Vpr as a result of fusion, after 2 hours of infection, is monitored by the enzymatic cleavage of CCF2-AM, a fluorogenic substrate of β-lactamase loaded in target cells. This assay was previously validated as being linear and able to detect differences in the 2-fold range [51], [52]. A typical experiment, with two different MOI, is represented in Fig. 4A. Using this system, we did not detect any significant effect of hyperthermia on viral fusion and access to the cytoplasm (Fig. 4B).

We then asked if HIV-1 reverse transcription was influenced by hyperthermia. Using an enzymatic *in vitro* cell-free assay, we monitored the reverse transcriptase (RT) activity of HIV-1 virions. Elevation of the temperature from 37°C to 39.5°C did not affect the enzymatic activity of RT (Fig. 4C), which is consistent with earlier studies [54], [55], [35]. To quantify reverse transcription in the context of infected cells, Jurkat cells were exposed to single-cycle virus, at two MOI. The levels of “early” and “late” RT products were determined by quantitative PCR at 8 hours p.i.. As expected, levels of viral DNA products correlated with the viral input, and the addition of Nevirapine (NVP), an RT inhibitor, almost completely blocked viral DNA synthesis (Fig. 4D). Hyperthermia had no effect on the levels of early or late RT products in infected cells, at 8 hours p.i. and other time points (Fig. 4D and not shown). Together, these results strongly suggest that hyperthermia acts after viral access to the cytoplasm and reverse transcription.

**Figure 5:**
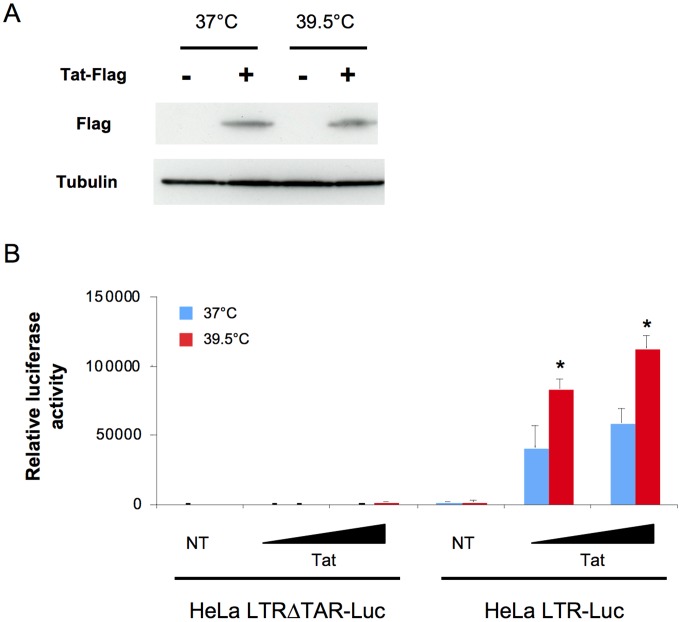
Hyperthermia enhances Tat-dependent LTR transcription. A: Levels of Tat in HeLa-LTR-Luc cells. Cells were transfected with 20 ng of Tat-Flag. After 4 hours at 37°C, cells were washed and grown at 37°C or 39.5°C for 48 hours. Tat-Flag and tubulin levels were assessed by Western Blot. B: LTR activity in HeLa cells at 37°C or 39.5°C. HeLa-LTR-Luc and HeLa-LTRΔTAR-Luc cells were transfected with 4 ng or 20 ng of Tat-Flag. After 4 hours at 37°C, cells were washed and grown at 37°C or 39.5°C. 48 hours later, luciferase levels were measured and normalized to protein concentration. Data are mean ± SD of 3 independent experiments. Statistical significance was assessed by the Mann-Withney test. p<0.05(*).

The lack of effect of a high temperature on LV-GFP transduction (Fig. 3) suggested a possible impact of hyperthermia on HIV-1 transcription. To test this hypothesis, we transfected HeLa cells carrying an integrated LTR-Luciferase cassette (HeLa LTR-Luc cells), with increasing amounts of a Tat-expressing plasmid (pcDNA-Tat-Flag). HeLa LTRΔTAR-Luc cells, unable to bind Tat, were used as a negative control. After 4 hours of incubation at 37°C, cells were washed to remove excess plasmids and transfection reagents, and grown at either 37°C or 39.5°C for 48 hours. We verified by Western Blot that Tat-Flag was expressed in equal amounts at 37°C and 39.5°C (Fig. 5A). Transactivation of the LTR was then assessed by measuring luciferase activity in cell lysates. Activity of the LTRΔTAR promoter was very low, with or without Tat, and hyperthermia had no effect on this residual activity (Fig. 5B). Tat efficiently stimulated the full length LTR, and this transactivation was significantly higher at 39.5°C than at 37°C (2 fold increase, Fig. 5B). The greater transactivation of the LTR at 39.5°C was not caused by a higher production of transfected Tat (Fig. 5A), by changes in Tat nuclear localization (not shown) nor by a *trans*-effect of secreted Tat by neighboring cells (Fig. S1B). In contrast to its action on the LTR promoter, hyperthermia did not significantly impact the activity of a CMV promoter, upon transfection of a pCMV-β-Galactosidase reporter plasmid (not shown). Therefore, a more potent activation of the LTR by Tat likely facilitates HIV-1 replication at 39.5°C.

### Hyperthermia boosts HIV-1 reactivation from latency in J-Lat cells

**Figure 6:**
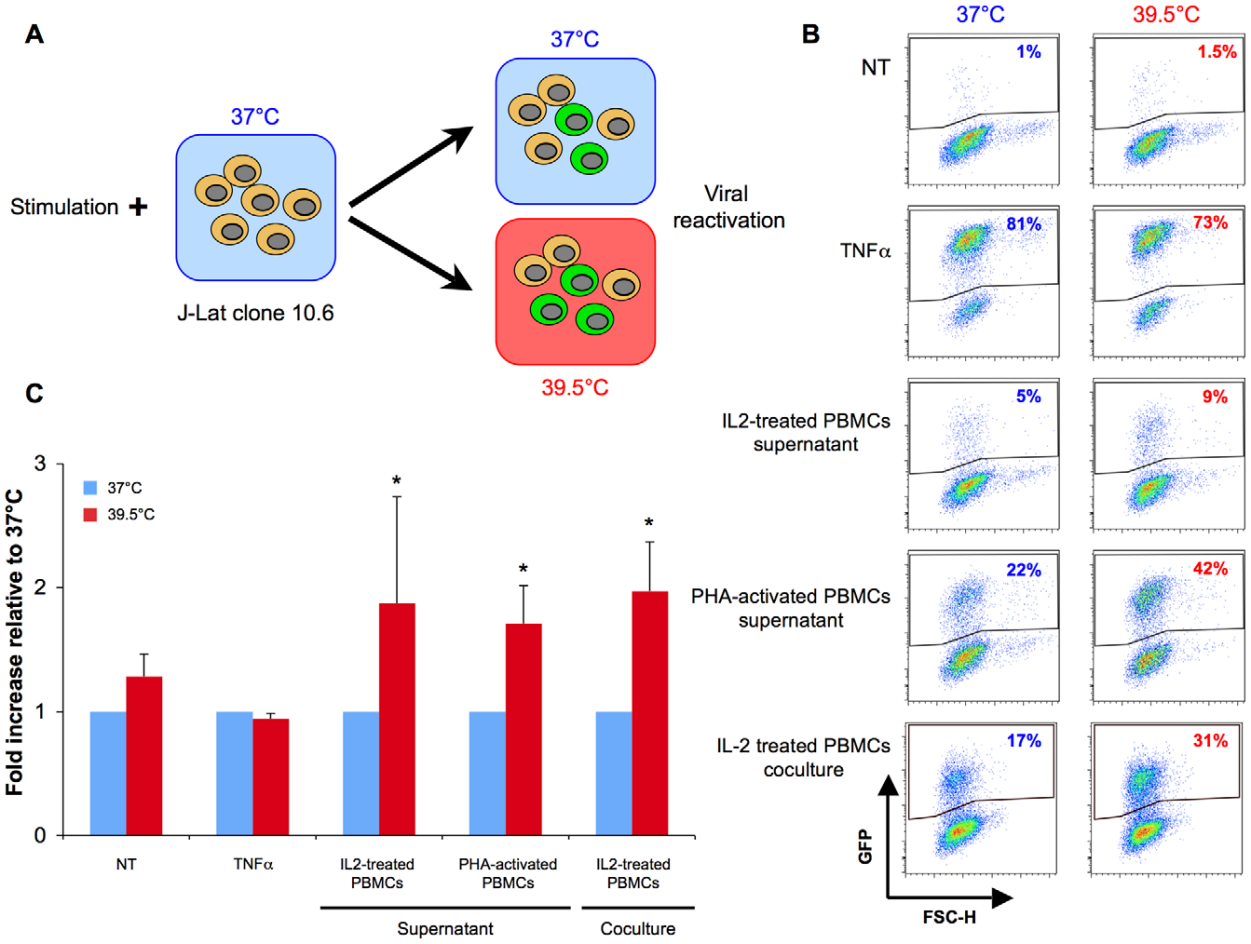
Hyperthermia increases viral reactivation in J-Lat 10.6 cells. A: Experimental outline. J-Lat 10.6 are Jurkat-derived cells carrying a latent, integrated, provirus, encoding *gfp* instead of *nef*. Stimulation of J-Lat 10.6 cells with TNFα triggers HIV-1 reactivation and GFP expression. B: Reactivation of J-Lat 10.6 cells with various stimuli. One representative experiment is shown. J-Lat 10.6 cells were treated with either TNFα, supernatants from IL-2 treated or PHA-activated PBMCs, or co-cultivated with IL-2-treated PBMCs for 48 hours at 37°C or 39.5°C. Viral reactivation was assessed by measuring GFP levels by flow cytometry. C: Mean ± SD of 4 independent experiments. Statistical significance was assessed by a paired t test. p<0.05(*).

The existence of latent HIV-1 reservoirs is a long-standing issue in the treatment of AIDS. The formation of these reservoirs is not prevented by Highly Active Antiretroviral Therapy (HAART) and drives viral re-emergence if therapy is stopped [56], [57], [58], [59], [60]. Reactivation from latency is a highly regulated process. Earlier studies outlined the impact of the site of integration, the role of pro-inflammatory cytokines, transcription factors like NFκB, and epigenetic modifications such as cytosine methylation [61], [62], [63], [64], [65]. We asked whether hyperthermia could trigger HIV-1 reactivation from latency, directly or in synergy with other stimuli. To this aim, we used the J-Lat 10.6 model [66]. Briefly, J-Lat 10.6 are Jurkat cells, carrying a latent, integrated provirus, where *env* is deleted and *nef* replaced by *gfp*. Without activation, GFP is not produced, but treatment with TNFα, PMA, or other molecules, induces HIV-1 reactivation and GFP expression. As outlined Fig. 6A, J-Lat 10.6 cells were exposed to various stimuli, and grown at 37°C or 39.5°C for 24 hours. Viral reactivation was then followed by measuring the appearance of GFP+ cells by flow-cytometry.

A representative experiment is shown Fig. 6B, and the mean of all experiments in Fig. 6C. Alone, hyperthermia was not sufficient to trigger viral reactivation (<2% of GFP+ cells at both temperatures). As a positive control, we used TNFα, which induced viral reactivation in up to 80% of J-Lat 10.6 cells (Fig. 6). Even used at sub-optimal concentrations, TNFα and PMA induced a similar reactivation at 37°C or 39.5°C (Fig. S2 and not shown), suggesting that these stimulators may be too strong to evidence any difference. We then used a more physiological stimulation, by exposing J-Lat 10.6 cells to conditioned medium from PBMCs. Supernatants from non stimulated PBMCs (but treated with IL-2 to avoid massive cell death), as well as from PHA-activated PBMCs, induced GFP expression in J-Lat 10.6 cells at 37°C (5% and 22% of GFP+ cells, respectively). Noteworthy, supernatants from PHA-activated PBMCs were more potent than that from non-stimulated PBMCs. This suggests that reactivation in J-Lat 10.6 cells is mediated by cytokines or other molecules that are up-regulated in activated PBMCs. Interestingly, PBMCs supernatants were more potent when J-Lat 10.6 cells were incubated at 39.5°C, with a significant 2-fold increase in the levels of GFP-expressing cells. We then directly co-cultivated J-Lat 10.6 cells with PBMCs. Co-culture with IL-2-treated PBMCs efficiently reactivated J-Lat cells, with up to 17% of GFP+ cells (Fig. 6B). Hyperthermia further increased this effect by 2-fold. Co-culture with PHA-activated PBMCs resulted in high levels of cell death (not shown).

There are different J-Lat clones, in which the extent of viral reactivation varies according to the viral integration site [62]. To check that the effect of hyperthermia on viral reactivation was not restricted to the 10.6 clone, we used J-Lat 6.3, J-Lat 8.4 and J-Lat 9.2 cells. These clones are less susceptible to reactivation than the 10.6 clone [62]. Indeed, we did not detect viral reactivation following exposure to PBMCs conditioned medium nor co-culture with PBMCs (not shown). However, stimulation with various doses of TNFα resulted in viral reactivation in J-Lat 6.3, J-Lat 8.4 and J-Lat 9.2 cells, at a weaker level than in J-Lat 10.6 (Fig. S2). Hyperthermia increased modestly but significantly the effect of TNFα in J-Lat 6.3, J-Lat 8.4 and J-Lat 9.2 cells. To achieve similar levels of GFP expression, the concentration of TNFα required at 37°C was two fold greater than at 39.5°C.

Altogether, these results show that PBMCs conditioned medium, as well as a direct co-culture with PBMCs, induce viral reactivation in the J-Lat model. Hyperthermia significantly enhances this phenomenon.

### Hsp90 mediates the stimulating effect of hyperthermia on HIV-1 infection

**Figure 7:**
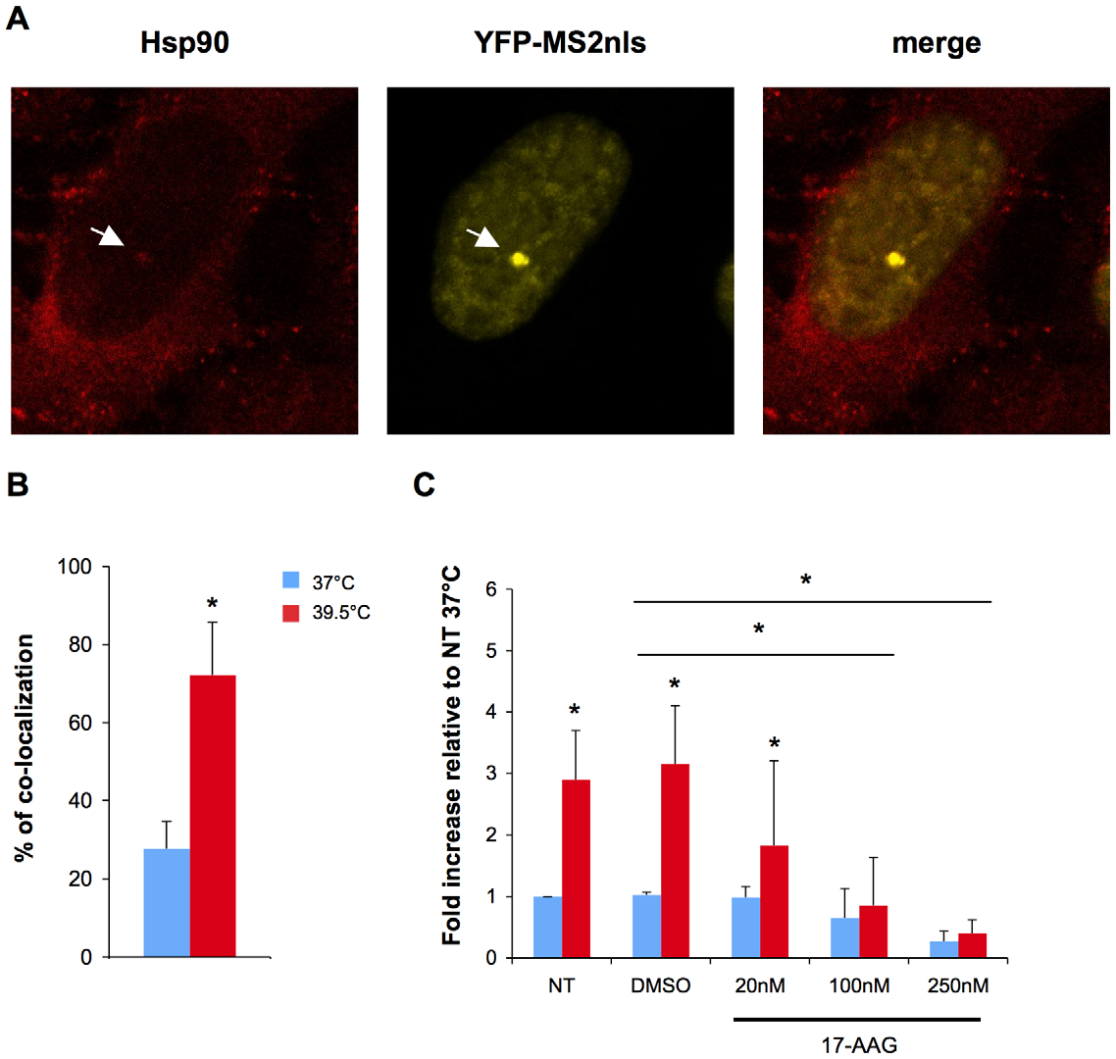
Role of Hsp90 during HIV-1 transcription and replication. A: Visualization of HIV transcription sites in U2OS HIVexo cells. One representative cell (at 37°C), displaying a co-localization of Hsp90 and nascent HIV RNA (followed with a YFP-MS2nls reporter protein) is shown. 2.5×10^5^ U2OS HIVexo cells were plated on coverslips and grown overnight at 37°C. Cells were transfected with 50 ng of Tat and 300 ng of YFP-MS2nls. After 4 hours, cells were washed and grown overnight at 37°C or 39.5°C. B: Mean ± SD of 3 independent experiments. Cells showing a transcription spot were scored for their co-localization with Hsp90. The percentage corresponds to cells in which HIV transcription sites co-localize with Hsp90. Statistical significance was assessed by an unpaired t test p<0.05(*). C: effect of the Hsp90 inhibitor 17-AAG on single-cycle HIV infection in P4C5 cells. Mean ± SD of 4 independent experiments is shown. 2×10^5^ P4C5 cells were infected with 30 ng Gag p24 of Δ*env*(VSV) for 2 hours at 37°C or 39.5°C, in presence of the indicated doses of 17-AAG. Cells were washed and grown at 37°C or 39.5°C at the indicated doses of 17-AAG. Cells were harvested 24 hours p.i. and Gag levels were assessed by flow cytometry. As a negative control, we used DMSO at a concentration corresponding to 250 nM 17-AAG.

Hsp90 may play a role during HIV-1 infection [48]. Chromatin immunoprecipitation experiments recently revealed that Hsp90 associates with the viral LTR and may regulate gene expression [48]. The underlying mechanisms are only partly understood, and may involve an effect of Hsp90 on chromatin remodeling, to facilitate transcription [48]. Moreover, the chaperone activity of Hsp90 has been reported to promote formation of a functional P-TEFb/Tat/TAR complex [67]. This prompted us to examine whether Hsp90 co-localizes with actively transcribing provirus in HIV-1-expressing cells. We used an original approach to directly visualize viral RNA in living cells. The technique takes advantage of an HIV-1 strain encoding an RNA which includes 24 binding sites for the phage MS2 protein (HIV_Exo_24×MS2) [68], [69], [70]. U2OS cells carrying an integrated HIV_Exo_24×MS2 genome (U2OS HIVexo) allow the visualization of nascent RNA from a single chromatin location [68], [69], [70]. Upon Tat expression, this RNA is synthesized and can be detected by specific high-affinity interaction with the YFP-MS2nls reporter protein. U2OS HIVexo cells were transfected with the YFP-MS2nls reporter, with or without a Tat-expressing plasmid. After an overnight incubation, the transcribing provirus and endogenous Hsp90 were both visualized by immunofluorescence. In the absence of Tat, YFP-MS2nls displayed a diffuse nuclear staining, whereas the Hsp90 signal was mostly detected in the cytoplasm with very little, if any, nuclear localization (not shown). With Tat, the nascent HIV RNA was detected as a single bright spot of YFP-MS2nls within the nucleus (Fig. 7A). Previous studies demonstrated that these spots represent true sites of viral transcription, rather than sites of HIV RNA sequestration [68]. At 37°C, Hsp90 co-localized with YFP-MS2nls in about 27% of the cells in which a nuclear YFP-MS2nls bright spot was visible (Fig. 7B), indicating Hsp90 can be recruited to the viral transcription site in HIV-infected cells. Hsp90 co-localization with HIV transcripts was significantly increased when cells were incubated overnight at 39.5°C, reaching 70% of the cells in which a viral transcription site was visible (Fig. 7B).

We determined further the role of Hsp90 during hyperthermia. We sought to silence Hsp90, but the extent of silencing achieved with various siRNAs or shRNAs was partial, precluding further analysis (not shown). We thus used a well-characterized pharmacological inhibitor of Hsp90, 17-AAG (also known as tanespimycin) [71]. This compound is a geldanamycin-derived molecule, inhibiting the ATPase activity of Hsp90 and blocking various functions of the heat shock protein [71], [72], [73]. Interestingly, 17-AAG is known to reduce HIV-1 replication, but its effects were studied so far at 37°C [48], [50]. We thus examined whether 17-AAG reversed the stimulating effect of hyperthermia on HIV-1 replication. To this aim, P4C5 cells were exposed to increasing doses of 17-AAG (20–250 nM) and infected with the single-cycle Δ*env*(VSV) virus. These 17-AAG concentrations were chosen because they did not induce obvious cytotoxicity (not shown). This is in agreement with a previous report indicating that the toxic concentration (CC_50_) is about 2 µM [50]. At 37°C, 17-AAG decreased infection, but this effect was modest, requiring 100–250 nM concentrations of the inhibitor (Fig. 7C). Interestingly, the compound was more active in hyperthermic cells, starting to decrease the infectivity enhancement at 20 nM, and abrogating this positive effect at 100 nM (Fig. 7C).

Therefore, Hsp90 is recruited to HIV-1 transcription sites, and this process occurs more efficiently at 39.5°C. The Hsp90 inhibitor 17-AAG significantly decreases the stimulating effect of hyperthermia on HIV-1 infection.

## Discussion

We report here a positive impact of hyperthermia on HIV-1 replication. Hyperthermia is known to enhance the functions of immune cells and to confer protection against pathogen infection [1], [2], [4], [5], Previous studies on temperature and HIV-1 mostly focused on chronically infected cell lines [31], [32] or used non-physiological heat shock treatment to study viral reactivation from latency (a few minutes at 42–45°C [33]). Here, we report that elevation of temperature to fever-like levels (39.5°C) stimulates HIV-1 replication in primary CD4+ T lymphocytes as well as in Hela and Jurkat cell lines. In single-cycle infection assays, hyperthermia increased HIV-1 infection by 2 to 7 fold. This stimulation was apparently not due to unspecific alterations of cellular metabolism, since cell growth, viability, or surface levels of various molecules were not significantly affected by hyperthermia.

To get insight into how hyperthermia stimulates HIV-1 replication, we compared the efficiency of various steps of the viral life cycle at 37°C and 39.5°C. Viral entry and fusion, measured by the Vpr-β-lactamase assay, were similar at the two temperatures. Enzymes have a range of conditions of pH, salt concentration, and temperature, in which they display optimal activity. We did not observe an effect of hyperthermia on reverse transcriptase, as both *in vitro* catalytic activity of the enzyme and the levels of viral DNA synthesis in infected cells were unchanged by temperature. We then examined the influence of temperature on the viral transcription step. Hyperthermia did not induce basal LTR activity without Tat. However, in the presence of Tat, hyperthermia lead to a significantly better transactivation of the LTR. This is in line with earlier reports, demonstrating that a transient heat shock at 42°C increases HIV-1 transcription in monocytic cells lines [31], [32]. Noteworthy, the activity of the CMV promoter was not increased at 39.5°C (not shown), suggesting that hyperthermia does not trigger a global increase of cellular transcription. Accordingly, the steady state levels of several cellular proteins (actin, CD4, ICAM-1, MHC-I, etc.) were apparently similar at normal and elevated temperatures.

To characterize the molecular mechanism by which hyperthermia up-regulates HIV-1 infection and transcription, we examined the role of Hsp90. This protein exerts diverse functions in normal and stressed cells, through its ATPase activity and its protein binding domain [72], [73]. It acts as a chaperone for many cellular proteins. Hsp90 assists folding, assembly, intracellular transport, maintenance and degradation of proteins, and regulates cell signaling and cell cycle [74], [75]. Hsp90 is involved in HIV-1 infection at 37°C, regulating viral gene expression [48]. Hsp90 also impacts the replication of other viral species, such as Human Cytomegalovirus, Influenza Virus, Flock House Virus and Hepatitis C Virus [76], [77], [78], [79]. We show here that the levels of Hsp90 are augmented at 39.5°C, in primary lymphocytes and other cells (Fig. 2 and not shown). By using an immunofluorescence technique allowing the visualization of nascent viral RNA in living cells, we demonstrate that, in presence of Tat, Hsp90 can be found in the nucleus, at HIV-1 transcription sites. This localization was rather infrequent at 37°C, but was significantly increased at 39.5°C (27% and 70% co-localization, respectively). Furthermore, 17-AAG, a pharmacological inhibitor of Hsp90, reversed the stimulating effect of hyperthermia on single-cycle infection in P4C5 cells. Altogether, these results point out for a previously uncharacterized role of Hsp90, facilitating HIV-1 transcription and replication at 39.5°C. It will be worth further dissecting how Hsp90 acts on viral transcription at this temperature. One can speculate that the chaperone protein may bind more efficiently to the P-TEFb/Tat/TAR transcription complex [67] and thus increase its activity, and/or may enhance chromatin modeling and accessibility to the viral promoter [48].

Mechanisms regulating HIV-1 gene expression are also involved in viral reactivation from latency [80]. We show here that the conditioned medium from PBMCs induced viral reactivation, in the J-Lat 10.6 model of latently infected T cells. Strikingly, reactivation was more pronounced at 39.5°C than at 37°C. Futures studies will help understanding which cytokines or other molecules produced by PBMCs mediate this effect. For instance, heat shock at 42°C is known to act in synergy with IL-6 to induce viral reactivation in a latently infected monocytic cell-line [31]. It will be of interest to compare the stimulating effect of IL-6 and other cytokines, at normal and fever-like temperatures, not only in J-Lat cells, but also in other models of viral latency (PBMCs from HAART-treated patients, or latently-infected, resting primary CD4+ T cells [81]).

In this study, we have focused our analysis of the effect of temperature on a few key steps of the viral life cycle. We demonstrate that hyperthermia globally facilitates viral replication. At 39.5°C, viral entry, fusion and reverse transcription occur normally, whereas Tat-mediated transactivation of the LTR is significantly more efficient. It has been previously reported that the activity of HIV-1 integrase and protease is not increased at 39.5°C [35]. This does not rule out the possibility that other steps of HIV-1 infection (nuclear import, selection of integration sites in the cellular genome, viral translation, assembly, release, etc.) might be positively or negatively modified at a fever-like temperature.

What is the physiological relevance of our observations? Patients treated with HAART and with controlled viremia can experience transient bursts of HIV-1 replication termed viral blips [82]. Furthermore, co-infections are frequent in HIV-1-positive individuals and are often associated with fever and acute illnesses [83]. For instance, *Plasmodium falciparum*, the causative agent of malaria, induces recurrent, strong episodes of fever lasting 2–3 days, which correlates with increased viral loads [84]. The origin of these viral blips, or of other more pronounced viral rebounds is likely multi-factorial. Our results suggest that fever may directly stimulate viral replication or reactivation from latent reservoirs, in association with other inflammatory or immunological events.

## Materials and Methods

### Cells, viruses, reagents

Jurkat (clone 20), MT4C5, J-Lat (clone 6.3, 8.4, 9.2 and 10.6), PBMCs and primary CD4+ T cells were grown in RPMI 1640 with Glutamax, supplemented with 10% heat-inactivated Fetal Bovine Serum (FBS) and antibiotics. HEK-293T, U2OS HIVexo [68], HeLa, HeLa Tat, and P4C5 cells were grown in Dulbecco modified Eagle medium (DMEM) supplemented with 10% heat-inactivated FBS and antibiotics. For P4C5 cells, a HeLa-derived cell line expressing CD4, CCR5, and harboring a LTR-*lacZ* reporter cassette, G418 (500 µg.mL^−1^, Sigma) and Hygromycin B (50 µg.mL^−1^, PAA) were added to the culture medium. HeLa expressing Tat were grown in presence of Methotrexate (2 µM, Sigma). Primary CD4+ T cells were purified from human peripheral blood by Ficoll centrifugation, followed by immunomagnetic selection (Miltenyi Biotec). The blood was provided by the EFS (Etablissement Français du Sang, the French Official Blood Bank). About 98% of cells were CD4+ CD3+. For activation, primary CD4+ T cells were treated with phytohemagglutinin (PHA, 1 µg.ml^−1^, Remel Europe LTD) for 24 hours and then cultured in interleukin 2 (IL-2)-containing medium (50 U.mL^−1^, Abcys). If not stated different, cells were grown at 37°C, 5% CO_2_. Cells grown at 39.5°C were cultivated in a distinct incubator. Temperature was monitored by 2 different thermometers. HIV-1 strains, including NL4-3 and NLAD8, were produced by calcium-phosphate transfection of HEK-293T cells. The primary isolate 132W [85] was produced by infection of MT4C5 cells. Vesicular stomatitis virus type G (VSV-G) pseudotyped viruses were generated by co-transfection of HEK-293T cells with the NL4-3 provirus and VSV-G expression plasmid (8∶1 ratio). For the production of Blam-Vpr containing viruses, HEK-293T cells were co-transfected with NL4-3 or NL4-3-F522Y proviruses along with a Blam-Vpr expression plasmid (3∶1 ratio), kindly provided by Warner Greene [52], [51]. NL4-3-F552Y provirus encodes a non-fusogenic gp120/g41 Env complex [86]. LV-GFP were produced by co-transfection of HEK-293T cells by the packaging plasmid (R8-2), the GFP plasmid (pTrip-GFP) and VSV-G expression plasmid (5∶5∶1 ratio). Nevirapine (NIH Catalog, n°4666, batch 01990) was used at 25 nM. 17-AAG (Enzo Life Sciences) was diluted in DMSO and used at the indicated concentrations.

### Intracellular and surface molecule stainings

Cell surface stainings were performed at 4°C for 30 min with the following monoclonal antibodies (mAbs): ICAM-1 (clone 1H4, Immunotools, dilution 1/20), CD11a (Immunotools, 1/10), CD18 (Immunotools, 1/10), CD69 (clone FN50, BD, 1/30), MHC-I (clone W632, Sigma, 1/100), HLA-A2 (clone BB7.2, BD, 1/50), CD4 (clone SK3, BD, 1/30), CXCR4 (clone 12G5, NIH AIDS Research and Reference reagent Program, 1/100), Goat anti-Mouse Alexa 647 (Invitrogen, 1/400). Isotype-matched mAbs were used as negative controls. Cells were fixed in PBS-paraformaldehyde (PFA, Sigma) 4% for 5 min. For quantification of Gag levels, cells were harvested at indicated time points and fixed in PBS-PFA 4% for 5 min. Cells were washed in PBS and stained for 30 min in PBS containing 1% BSA (Sigma), 50 µg.mL^−1^ saponin (Sigma), and the anti-Gag antibody (clone KC57, 1/500, Beckman Coulter). Fluorescence was assessed by flow cytometry on a FacsCanto II (BD).

### Western Blot

Cells transfected with 20 ng of Tat-Flag were lysed with passive lysis buffer (Promega) and probed by Western Blot for Tubulin (T9026, Sigma, 1/5000) and Tat (Flag antibody, F3165, Sigma, 1/5000). Cells cultured for 8 hours at 37°C or 39.5°C were lysed in PBS Triton 1% in presence of a proteases inhibitors cocktail (Roche) and probed by Western Blot for Actin (clone AC-15, Sigma, 1/5000), Hsp70 (clone W27, Santa Cruz, 1/200) and Hsp90 (clone 68, BD, 1/1000, kindly provided by Jean-Michel Heard).

### Single-cycle infection assays

One day before infection, 8×10^4^ P4C5 cells were plated in 96-well plates. Cells were infected in triplicate with 1 or 5 ng Gag p24 per well. Cells were lysed in PBS 0.1% NP40 5 mM MgCl_2_ 36 hours post-infection, and incubated at room temperature with Chlorophenol Red-β-D-Galactopyranoside (CPRG, Roche) at a final concentration of 3.65 mg.mL^−1^. 570 nm OD was measured every 15 min. Jurkat cells were infected with 5, 20 or 100 ng Gag p24/mL/10^6^ at 37°C or 39.5°C for 2 hours. Cells were washed in PBS and grown at either 37°C or 39.5°C. Gag or GFP levels were assessed by flow cytometry 24 hours p.i.

### Viral replication assays in Jurkat and primary CD4+ T cells

Jurkat or primary CD4+ T cells (1×10^6^) were infected with the indicated doses of NL4-3 for 2 hours at 37°C or 39.5°C, washed once in PBS, and grown at 37°C or 39.5°C. Medium was changed every 2–3 days and samples were harvested at the indicated time points. Gag levels were assessed by flow cytometry. To combine results from independent experiments, we have measured the area under the curve at 37°C and 39.5°C. In Jurkat cells, there is no real peak of viral replication, since most of the cells (>80%) may get infected, and then die. In primary CD4+ T cells, a peak of infected cells may be detected, probably because a fraction of the cells is not sensitive to infection. We have thus calculated the area under the curve for all time points in Jurkat, and for time points occurring until the appearance of the peak at 39.5°C in primary CD4+ T cells.

### Luciferase assay

HeLa LTR-Luc cells contain a single copy of integrated HIV-1 LTR-luciferase reporter construct [87] and were transfected with 4 ng or 20 ng of pcDNA-Tat-Flag completed to 2 µg pcDNA3.1, or pcDNA3.1 alone, using JetPEI reagent (Polyplus) in 6 well plates. After 4 hours at 37°C, cells were washed and grown at 37°C or 39.5°C. 48 hours after incubation, cells were lysed using Passive Lysis Buffer (Promega) and luciferase activity was measured according to the manufacturer's protocol (Promega). Luciferase activity was normalized to protein concentration using Bradford assay (Biorad).

### Blam-Vpr assay

Viral entry was assessed by a test adapted from Cavrois *et al.* [51], [52]. Briefly, 5 or 100 ng Gag p24 of ultracentrifuged virions containing the Blam-Vpr fusion protein were used to infect 1.5×10^5^ Jurkat cells at 37°C or 39.5°C in a minimal volume (100 µL), in presence of 10 µM Hepes and 2 µg.mL^−1^ DEAE Dextran (Sigma). After 2 hours, cells were washed in cold CO_2_-independent medium (Invitrogen), without FBS, resuspended in cold CO_2_-independent medium supplemented with 10% FBS and incubated with the CCF2-AM substrate (CCF2-AM kit, Invitrogen), in the presence of 1.8 mM Probenecid (Sigma), for 2 hours. Cells were extensively washed in cold CO_2_-independent medium and fixed in PBS-PFA 4% for 5 min. The cleaved CCF2-AM fluorescence (excitation at 405 nm, emission at 450 nm) was then immediately measured by flow cytometry using the DAPI channel, on a FacsCanto II (BD).

### *In vitro* RT enzymatic assay

NL4-3 virions were lysed in PBS NP40 0.08% and incubated with Retrosys RT Activity Kit (Innovagen) substrates for 1 or 3 hours, according to the manufacturer's indications. RT activity was measured with a alcaline phosphatase readout (405 nm OD).

### Quantification of HIV-1 DNA

Jurkat cells were infected with 50 or 250 ng Gag p24 at 37°C or 39.5°C with NL4-3 Δ*env*(VSV). After 2 hours, cells were washed and grown at 37°C or 39.5°C. Cells were harvested 8 hours p.i. and lysed in AL Buffer (Qiagen) in presence of Proteinase K (Qiagen) for 1 hour at 56°C. Total DNA was extracted from by phenol-chloroform extraction and ethanol precipitation. To remove all traces of plasmidic DNA, samples were treated with DpnI Fast Digest (Fermentas) for 15 min at 37°C. To avoid inhibition of the PCR reaction by DpnI reaction buffer, samples were diluted 40 times in pure water (Gibco). Early RT products (amplicon length: 183 bp) were quantified by real-time PCR with the primers M667 (GGC TAA CTA GGG AAC CCA CTG) and AASM (GCT AGA GAT TTT CCA CAC TGA CTA A) using the the following program: 35 cycles 10 s 95°C, 10 s 57°C, 15 s 72°C. For late RT products (amplicon length: 200 bp), we used the primers M667 and M661 (CCT GCG TCG AGA GAG CTC CTC TGG), with a slightly different PCR program (35 cycles, 10 s 95°C, 15 s 57°C, 15 s 72°C). The number of cells was calculated by quantification of genomic GAPDH (amplicon length: 303 bp), using the same program as late RT products and the following primers: forward (GGG AAA CTG TGG CGT GAT) and reverse (GGA GGA GTG GGT GTC GTT).

### Viral reactivation assay

J-Lat clones 6.3, 8.4, 9.2 and 10.6, kindly provided by Stéphane Emiliani, have been previously described [66]. Briefly, J-Lat are Jurkat cells with a latent, integrated, and *env*-deleted provirus, encoding *gfp* instead of *nef*. Stimulation of J-Lat cells with recombinant TNFα (Peprotech) or other molecules such as PMA triggers HIV-1 reactivation. PBMCs were isolated from peripheral blood, PHA-activated or treated with IL-2 for 3 days. Supernatants were then collected and used to stimulate J-Lat cells. Serial dilutions of supernatants were tested, as raw supernatants were sometimes toxic. For co-cultures experiments, PBMCs were isolated from peripheral blood, treated for 3 days with IL-2, and co-cultivated with Far-Red labelled J-Lat 10.6 cells at a 1∶4 ratio in IL-2-containing medium. 48 hours after stimulation, cells were harvested, fixed in PBS-PFA 4%. Viral reactivation was followed by appearance of GFP+ J-Lat cells by flow-cytometry.

### Localization of HIV-1 transcription sites by immunofluorescence

U2OS HIVexo cells were transfected with a Tat-expressing plasmid to stimulate viral gene expression. The transcribing provirus was detected by MS2 tagging, Hsp90 was detected by immunofluorescence with a monoclonal antibody. YFP-MS2nls, yellow fluorescent protein fused to MS2 with a nuclear localizing signal. U2OS HIVexo cells and plasmids for Tat and EYFP-MS2nls expression were described previously [68]. 2.5×10^5^ cells were plated on coverslips and grown overnight at 37°C. Next day, cells were transfected (Lipofectamine LTX, Invitrogen) with plasmids expressing Tat (50 ng) and YFP-MS2nls (300 ng). After 4 hours cells were washed and shifted to an incubator at 39.5°C in medium containing 25 mM Hepes for 20 hours. Control cells were kept at 37°C also with 25 mM Hepes in sealed flasks. After treatment, cells were fixed and treated for immunofluorescence essentially as described [88] with a mouse mAb (H90-10, Abcam, 1/500). Cells showing a transcription spot were scored for their co-localization with Hsp90 on a confocal microscope Zeiss 510 META.

### Statistical analyses

Statistical tests were performed with GraphPad Prism 5.

## Supporting Information

Figure S1 **Hyperthermia does not alter half-life of virions and does not promote Tat bystander effect.** A: Half life of viral preparations at 37°C and 39.5°C. Viral preparations were incubated in medium at 37°C or 39.5°C for the indicated times (from 15 min to 24 hours). P4C5 cells were infected in triplicates with 1 ng Gag p24 and grown at 37°C. Infection was assessed 36 hours p.i. by measuring β-Galactosidase activity (570 nm OD). The background OD, corresponding to non-infected cells is indicated in grey. The half-life was calculated as 50% of the initial infectivity. One of three representative experiments is shown. Data are mean ± SD of triplicates. B: Co-culture of HeLa Tat and P4C5 cells. 8000 P4C5 cells were co-cultured with 1000, 2000, 4000 or 8000 HeLa Tat cells at 37°C or 39.5°C. After 16 hours of co-culture, cells were lysed and the *trans*-effect of secreted Tat on the LTR promoter was assessed by measuring β-Galactosidase activity (OD). HeLa Tat cells express functional Tat protein, as assessed by transfecting a LTR-luciferase reporter plasmid (not shown). One of three representative experiments is shown.
(TIF)

Figure S2 **Hyperthermia enhances TNFα-mediated viral reactivation in several J-Lat clones.** J-Lat 6.3, J-Lat 8.4, J-Lat 9.2 or J-Lat 10.6 cells (1×10^5^ per well) were stimulated at 37°C or 39.5°C with the following doses of recombinant TNFα: 0.5; 5 or 10 ng.mL^−1^. Viral reactivation was assessed 48 hours later by measuring GFP levels by flow cytometry. Data are mean ± SD of 4 independent experiments. Statistical significance was assessed by a paired t test. p<0.05(*).
(TIF)

## References

[1] Ostberg JR, Repasky EA (2006). Emerging evidence indicates that physiologically relevant thermal stress regulates dendritic cell function.. Cancer Immunol Immunother.

[2] Hasday JD, Fairchild KD, Shanholtz C (2000). The role of fever in the infected host.. Microbes Infect.

[3] Evans SS, Wang WC, Bain MD, Burd R, Ostberg JR (2001). Fever-range hyperthermia dynamically regulates lymphocyte delivery to high endothelial venules.. Blood.

[4] Ostberg JR, Dayanc BE, Yuan M, Oflazoglu E, Repasky EA (2007). Enhancement of natural killer (NK) cell cytotoxicity by fever-range thermal stress is dependent on NKG2D function and is associated with plasma membrane NKG2D clustering and increased expression of MICA on target cells.. J Leukoc Biol.

[5] van Bruggen I, Robertson TA, Papadimitriou JM (1991). The effect of mild hyperthermia on the morphology and function of murine resident peritoneal macrophages.. Exp Mol Pathol.

[6] Ishida Y, Hiraki A, Hirayama E, Koga Y, Kim J (2002). Temperature-sensitive viral infection: inhibition of hemagglutinating virus of Japan (Sendai virus) infection at 41 degrees.. Intervirology.

[7] De Marco A, Santoro MG (1993). Antiviral effect of short hyperthermic treatment at specific stages of vesicular stomatitis virus replication cycle.. J Gen Virol.

[8] Virgilio PL, Godinho-Netto MC, Carvalho Mda G (1997). Previous heat shock treatment inhibits Mayaro virus replication in human lung adenocarcinoma (A549) cells.. Res Virol.

[9] Chavez-Salinas S, Ceballos-Olvera I, Reyes-Del Valle J, Medina F, Del Angel RM (2008). Heat shock effect upon dengue virus replication into U937 cells.. Virus Res.

[10] Lopez C, Fitzgerald PA, Siegal FP (1983). Severe acquired immune deficiency syndrome in male homosexuals: diminished capacity to make interferon-alpha in vitro associated with severe opportunistic infections.. J Infect Dis.

[11] Zerbini M, Musiani M, La Placa M (1985). Effect of heat shock on Epstein-Barr virus and cytomegalovirus expression.. J Gen Virol.

[12] Kaper JM, Geletka LM, Wu GS, Tousignant ME (1995). Effect of temperature on cucumber mosaic virus satellite-induced lethal tomato necrosis is helper virus strain dependent.. Arch Virol.

[13] Kido K, Tanaka C, Mochizuki T, Kubota K, Ohki T (2008). High temperatures activate local viral multiplication and cell-to-cell movement of Melon necrotic spot virus but restrict expression of systemic symptoms.. Phytopathology.

[14] Modjarrad K, Vermund SH (2010). Effect of treating co-infections on HIV-1 viral load: a systematic review.. Lancet Infect Dis.

[15] McShane H (2005). Co-infection with HIV and TB: double trouble.. Int J STD AIDS.

[16] Idemyor V (2007). Human immunodeficiency virus (HIV) and malaria interaction in sub-Saharan Africa: the collision of two Titans.. HIV Clin Trials.

[17] Barnabas RV, Webb EL, Weiss HA, Wasserheit JN (2011). The role of coinfections in HIV epidemic trajectory and positive prevention: a systematic review and meta-analysis.. AIDS.

[18] Kiyingi HS, Egwang TG, Nannyonga M (2010). Prolonged elevation of viral loads in HIV-1-infected children in a region of intense malaria transmission in Northern Uganda: a prospective cohort study.. Pan Afr Med J.

[19] Bentwich Z (2003). Concurrent infections that rise the HIV viral load.. J HIV Ther.

[20] Collins KR, Quinones-Mateu ME, Toossi Z, Arts EJ (2002). Impact of tuberculosis on HIV-1 replication, diversity, and disease progression.. AIDS Rev.

[21] Toossi Z (2003). Virological and immunological impact of tuberculosis on human immunodeficiency virus type 1 disease.. J Infect Dis.

[22] Decrion AZ, Dichamp I, Varin A, Herbein G (2005). HIV and inflammation.. Curr HIV Res.

[23] McMichael AJ, Borrow P, Tomaras GD, Goonetilleke N, Haynes BF (2010). The immune response during acute HIV-1 infection: clues for vaccine development.. Nat Rev Immunol.

[24] Haase AT (2010). Targeting early infection to prevent HIV-1 mucosal transmission.. Nature.

[25] Brenchley JM, Silvestri G, Douek DC (2010). Nonprogressive and progressive primate immunodeficiency lentivirus infections.. Immunity.

[26] Cameron PU, Saleh S, Sallmann G, Solomon A, Wightman F (2010). Establishment of HIV-1 latency in resting CD4+ T cells depends on chemokine-induced changes in the actin cytoskeleton.. Proc Natl Acad Sci U S A.

[27] Poli G, Bressler P, Kinter A, Duh E, Timmer WC (1990). Interleukin 6 induces human immunodeficiency virus expression in infected monocytic cells alone and in synergy with tumor necrosis factor alpha by transcriptional and post-transcriptional mechanisms.. J Exp Med.

[28] Poli G, Kinter AL, Fauci AS (1994). Interleukin 1 induces expression of the human immunodeficiency virus alone and in synergy with interleukin 6 in chronically infected U1 cells: inhibition of inductive effects by the interleukin 1 receptor antagonist.. Proc Natl Acad Sci USA.

[29] Fauci AS (1996). Host factors and the pathogenesis of HIV-induced disease.. Nature.

[30] Kedzierska K, Crowe SM (2001). Cytokines and HIV-1: interactions and clinical implications.. Antivir Chem Chemother.

[31] Stanley SK, Bressler PB, Poli G, Fauci AS (1990). Heat shock induction of HIV production from chronically infected promonocytic and T cell lines.. J Immunol.

[32] Hashimoto K, Baba M, Gohnai K, Sato M, Shigeta S (1996). Heat shock induces HIV-1 replication in chronically infected promyelocyte cell line OM10.1.. Arch Virol.

[33] Furlini G, Re MC, Musiani M, Zerbini ML, La Placa M (1990). Enhancement of HIV-1 marker detection in cell cultures treated with mild heat-shock.. Microbiologica.

[34] Harada S, Yusa K, Monde K, Akaike T, Maeda Y (2005). Influence of membrane fluidity on human immunodeficiency virus type 1 entry.. Biochem Biophys Res Commun.

[35] Wiskerchen M, Muesing MA (1995). Identification and characterization of a temperature-sensitive mutant of human immunodeficiency virus type 1 by alanine scanning mutagenesis of the integrase gene.. J Virol.

[36] Calderwood SK (2010). Heat shock proteins in breast cancer progression–a suitable case for treatment?. Int J Hyperthermia.

[37] Allegra A, Sant'antonio E, Penna G, Alonci A, D'Angelo A (2011). Novel therapeutic strategies in multiple myeloma: role of the heat shock protein inhibitors.. Eur J Haematol.

[38] Ciocca DR, Fanelli MA, Cuello-Carrion FD, Castro GN (2010). Heat shock proteins in prostate cancer: from tumorigenesis to the clinic.. Int J Hyperthermia.

[39] Torigoe T, Tamura Y, Sato N (2009). Heat shock proteins and immunity: application of hyperthermia for immunomodulation.. Int J Hyperthermia.

[40] Bolhassani A, Rafati S (2008). Heat-shock proteins as powerful weapons in vaccine development.. Expert Rev Vaccines.

[41] Brenner BG, Wainberg MA (1999). Heat shock protein-based therapeutic strategies against human immunodeficiency virus type 1 infection.. Infect Dis Obstet Gynecol.

[42] Gurer C, Cimarelli A, Luban J (2002). Specific incorporation of heat shock protein 70 family members into primate lentiviral virions.. J Virol.

[43] Agostini I, Popov S, Li J, Dubrovsky L, Hao T (2000). Heat-shock protein 70 can replace viral protein R of HIV-1 during nuclear import of the viral preintegration complex.. Exp Cell Res.

[44] Bukrinsky M, Zhao Y (2004). Heat-shock proteins reverse the G2 arrest caused by HIV-1 viral protein R.. DNA Cell Biol.

[45] Iordanskiy S, Zhao Y, DiMarzio P, Agostini I, Dubrovsky L (2004). Heat-shock protein 70 exerts opposing effects on Vpr-dependent and Vpr-independent HIV-1 replication in macrophages.. Blood.

[46] Iordanskiy S, Zhao Y, Dubrovsky L, Iordanskaya T, Chen M (2004). Heat shock protein 70 protects cells from cell cycle arrest and apoptosis induced by human immunodeficiency virus type 1 viral protein R.. J Virol.

[47] Kumar M, Rawat P, Khan SZ, Dhamija N, Chaudhary P (2011). Reciprocal regulation of human immunodeficiency virus-1 gene expression and replication by heat shock proteins 40 and 70.. J Mol Biol.

[48] Vozzolo L, Loh B, Gane PJ, Tribak M, Zhou L (2010). Gyrase B inhibitor impairs HIV-1 replication by targeting Hsp90 and the capsid protein.. J Biol Chem.

[49] Rawat P, Mitra D (2011). Cellular heat shock factor 1 positively regulates human immunodeficiency virus-1 gene expression and replication by two distinct pathways.. Nucleic Acids Res.

[50] Joshi P, Stoddart CA (2011). Impaired infectivity of ritonavir-resistant HIV is rescued by heat shock protein 90AB1.. J Biol Chem.

[51] Cavrois M, De Noronha C, Greene WC (2002). A sensitive and specific enzyme-based assay detecting HIV-1 virion fusion in primary T lymphocytes.. Nat Biotechnol.

[52] Cavrois M, Neidleman J, Bigos M, Greene WC (2004). Fluorescence resonance energy transfer-based HIV-1 virion fusion assay.. Methods Mol Biol.

[53] Casartelli N, Sourisseau M, Feldmann J, Guivel-Benhassine F, Mallet A (2010). Tetherin restricts productive HIV-1 cell-to-cell transmission.. PLoS Pathog.

[54] Piskareva O, Denmukhametova S, Schmatchenko V (2003). Functional reverse transcriptase encoded by the human LINE-1 from baculovirus-infected insect cells.. Protein Expr Purif.

[55] Huber HE, McCoy JM, Seehra JS, Richardson CC (1989). Human immunodeficiency virus 1 reverse transcriptase. Template binding, processivity, strand displacement synthesis, and template switching.. J Biol Chem.

[56] Chun TW, Stuyver L, Mizell SB, Ehler LA, Mican JA (1997). Presence of an inducible HIV-1 latent reservoir during highly active antiretroviral therapy.. Proc Natl Acad Sci U S A.

[57] Finzi D, Hermankova M, Pierson T, Carruth LM, Buck C (1997). Identification of a reservoir for HIV-1 in patients on highly active antiretroviral therapy.. Science.

[58] Lafeuillade A, Stevenson M (2011). The search for a cure for persistent HIV reservoirs.. AIDS Rev.

[59] Marcello A (2006). Latency: the hidden HIV-1 challenge.. Retrovirology.

[60] Chun TW, Davey RT, Engel D, Lane HC, Fauci AS (1999). Re-emergence of HIV after stopping therapy.. Nature.

[61] Moriuchi H, Moriuchi M, Mizell SB, Ehler LA, Fauci AS (2000). In vitro reactivation of human immunodeficiency virus 1 from latently infected, resting CD4+ T cells after bacterial stimulation.. J Infect Dis.

[62] Williams SA, Chen LF, Kwon H, Fenard D, Bisgrove D (2004). Prostratin antagonizes HIV latency by activating NF-kappaB.. J Biol Chem.

[63] Dieudonne M, Maiuri P, Biancotto C, Knezevich A, Kula A (2009). Transcriptional competence of the integrated HIV-1 provirus at the nuclear periphery.. EMBO J.

[64] Gallastegui E, Millan-Zambrano G, Terme JM, Chavez S, Jordan A (2011). Chromatin reassembly factors are involved in transcriptional interference promoting HIV latency.. J Virol.

[65] Kauder SE, Bosque A, Lindqvist A, Planelles V, Verdin E (2009). Epigenetic regulation of HIV-1 latency by cytosine methylation.. PLoS Pathog.

[66] Jordan A, Bisgrove D, Verdin E (2003). HIV reproducibly establishes a latent infection after acute infection of T cells in vitro.. EMBO J.

[67] O'Keeffe B, Fong Y, Chen D, Zhou S, Zhou Q (2000). Requirement for a kinase-specific chaperone pathway in the production of a Cdk9/cyclin T1 heterodimer responsible for P-TEFb-mediated tat stimulation of HIV-1 transcription.. J Biol Chem.

[68] Boireau S, Maiuri P, Basyuk E, de la Mata M, Knezevich A (2007). The transcriptional cycle of HIV-1 in real-time and live cells.. J Cell Biol.

[69] Maiuri P, Knezevich A, Bertrand E, Marcello A (2011). Real-time imaging of the HIV-1 transcription cycle in single living cells.. Methods.

[70] Kula A, Guerra J, Knezevich A, Kleva D, Myers MP (2011). Characterization of the HIV-1 RNA associated proteome identifies Matrin 3 as a nuclear cofactor of Rev function.. Retrovirology.

[71] Roe SM, Prodromou C, O'Brien R, Ladbury JE, Piper PW (1999). Structural basis for inhibition of the Hsp90 molecular chaperone by the antitumor antibiotics radicicol and geldanamycin.. J Med Chem.

[72] Obermann WM, Sondermann H, Russo AA, Pavletich NP, Hartl FU (1998). In vivo function of Hsp90 is dependent on ATP binding and ATP hydrolysis.. J Cell Biol.

[73] Panaretou B, Prodromou C, Roe SM, O'Brien R, Ladbury JE (1998). ATP binding and hydrolysis are essential to the function of the Hsp90 molecular chaperone in vivo.. EMBO J.

[74] Richardson PG, Mitsiades CS, Laubach JP, Lonial S, Chanan-Khan AA (2011). Inhibition of heat shock protein 90 (HSP90) as a therapeutic strategy for the treatment of myeloma and other cancers.. Br J Haematol.

[75] Yamaki H, Nakajima M, Shimotohno KW, Tanaka N (2011). Molecular basis for the actions of Hsp90 inhibitors and cancer therapy.. J Antibiot (Tokyo).

[76] Basha W, Kitagawa R, Uhara M, Imazu H, Uechi K (2005). Geldanamycin, a potent and specific inhibitor of Hsp90, inhibits gene expression and replication of human cytomegalovirus.. Antivir Chem Chemother.

[77] Nakagawa S, Umehara T, Matsuda C, Kuge S, Sudoh M (2007). Hsp90 inhibitors suppress HCV replication in replicon cells and humanized liver mice.. Biochem Biophys Res Commun.

[78] Chase G, Deng T, Fodor E, Leung BW, Mayer D (2008). Hsp90 inhibitors reduce influenza virus replication in cell culture.. Virology.

[79] Castorena KM, Weeks SA, Stapleford KA, Cadwallader AM, Miller DJ (2007). A functional heat shock protein 90 chaperone is essential for efficient flock house virus RNA polymerase synthesis in Drosophila cells.. J Virol.

[80] Trono D, Van Lint C, Rouzioux C, Verdin E, Barre-Sinoussi F (2010). HIV persistence and the prospect of long-term drug-free remissions for HIV-infected individuals.. Science.

[81] Yang HC (2011). Primary cell models of HIV latency.. Curr Opin HIV AIDS.

[82] Rong L, Perelson AS (2009). Modeling HIV persistence, the latent reservoir, and viral blips.. J Theor Biol.

[83] Sulkowski MS, Chaisson RE, Karp CL, Moore RD, Margolick JB (1998). The effect of acute infectious illnesses on plasma human immunodeficiency virus (HIV) type 1 load and the expression of serologic markers of immune activation among HIV-infected adults.. J Infect Dis.

[84] Kublin JG, Patnaik P, Jere CS, Miller WC, Hoffman IF (2005). Effect of Plasmodium falciparum malaria on concentration of HIV-1-RNA in the blood of adults in rural Malawi: a prospective cohort study.. Lancet.

[85] Vendrame D, Sourisseau M, Perrin V, Schwartz O, Mammano F (2009). Partial inhibition of human immunodeficiency virus replication by type I interferons: impact of cell-to-cell viral transfer.. J Virol.

[86] Nobile C, Moris A, Porrot F, Sol-Foulon N, Schwartz O (2003). Inhibition of human immunodeficiency virus type 1 Env-mediated fusion by DC-SIGN.. J Virol.

[87] Treand C, du Chene I, Bres V, Kiernan R, Benarous R (2006). Requirement for SWI/SNF chromatin-remodeling complex in Tat-mediated activation of the HIV-1 promoter.. EMBO J.

[88] Marcello A, Ferrari A, Pellegrini V, Pegoraro G, Lusic M (2003). Recruitment of human cyclin T1 to nuclear bodies through direct interaction with the PML protein.. EMBO J.

