# Supplementary figures and images for: Hyperthermia Stimulates HIV-1 Replication
Source: PLoS Pathog. 2012 Jul 12;8(7):e1002792. doi: 10.1371/journal.ppat.1002792 (PMC3395604; doi:10.1371/journal.ppat.1002792)

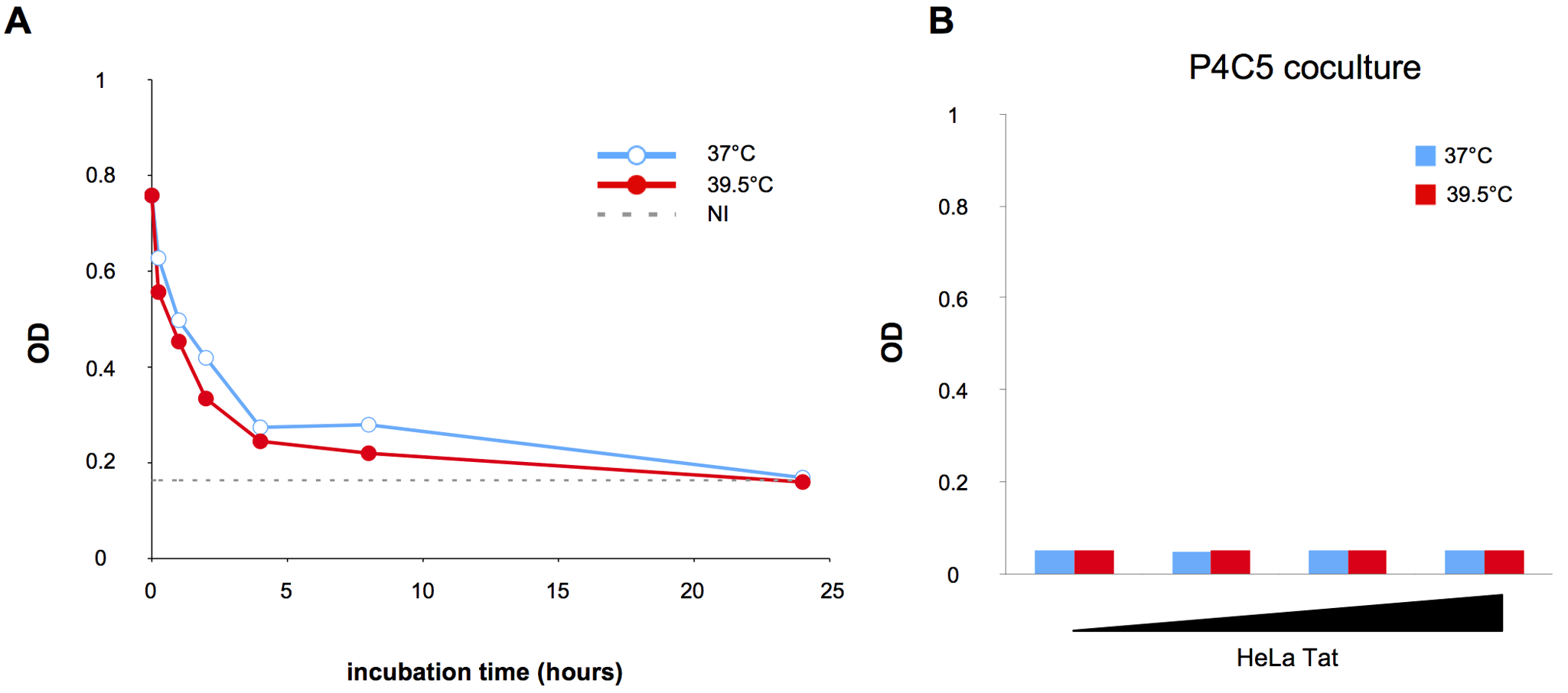

Supplement: Figure S1 — Hyperthermia does not alter half-life of virions and does not promote Tat bystander effect. A: Half life of viral preparations at 37°C and 39.5°C. Viral preparations were incubated in medium at 37°C or 39.5°C for the indicated times (from 15 min to 24 hours). P4C5 cells were infected in triplicates with 1 ng Gag p24 and grown at 37°C. Infection was assessed 36 hours p.i. by measuring β-Galactosidase activity (570 nm OD). The background OD, corresponding to non-infected cells is indicated in grey. The half-life was calculated as 50% of the initial infectivity. One of three representative experiments is shown. Data are mean ± SD of triplicates. B: Co-culture of HeLa Tat and P4C5 cells. 8000 P4C5 cells were co-cultured with 1000, 2000, 4000 or 8000 HeLa Tat cells at 37°C or 39.5°C. After 16 hours of co-culture, cells were lysed and the trans-effect of secreted Tat on the LTR promoter was assessed by measuring β-Galactosidase activity (OD). HeLa Tat cells express functional Tat protein, as assessed by transfecting a LTR-luciferase reporter plasmid (not shown). One of three representative experiments is shown. (TIF) [file ppat.1002792.s001.tif]

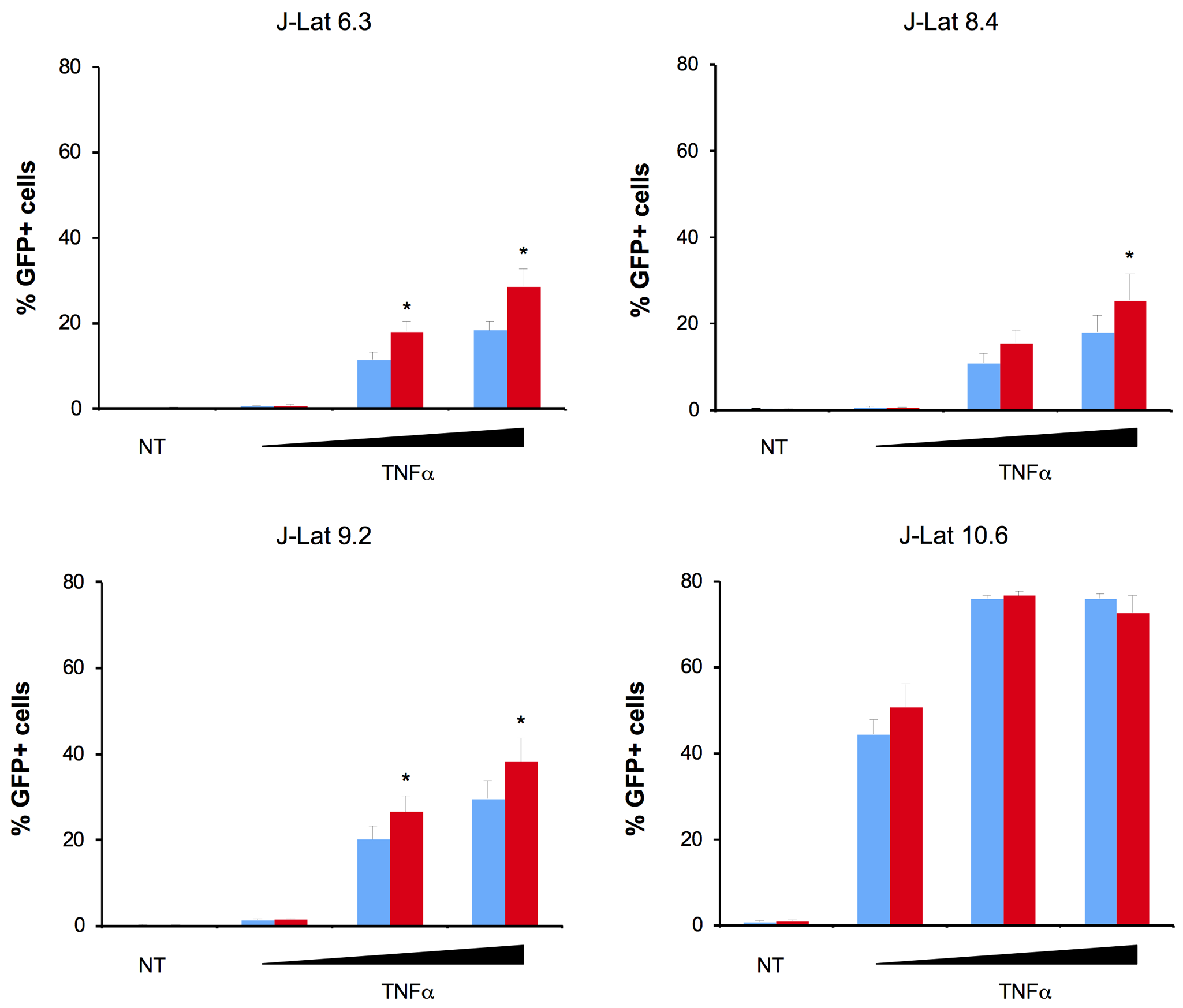

Supplement: Figure S2 — Hyperthermia enhances TNFα-mediated viral reactivation in several J-Lat clones. J-Lat 6.3, J-Lat 8.4, J-Lat 9.2 or J-Lat 10.6 cells (1×10^5^ per well) were stimulated at 37°C or 39.5°C with the following doses of recombinant TNFα: 0.5; 5 or 10 ng.mL−1. Viral reactivation was assessed 48 hours later by measuring GFP levels by flow cytometry. Data are mean ± SD of 4 independent experiments. Statistical significance was assessed by a paired t test. p<0.05(*). (TIF) [file ppat.1002792.s002.tif]
